# Beverage patterns, blood pressure, and proteinuria among West Africans with chronic kidney disease: a cross-sectional analysis of the diet, CKD, and apolipoprotein L1 study

**DOI:** 10.3389/fnut.2026.1724375

**Published:** 2026-02-06

**Authors:** Rayan Djelmami-Hani, Tolulope Adebile, Runqi Zhao, Edward Kwakyi, Oluwatoyin Amira, Bolanle Omotoso, Manmak Mamven, Yemi Raheem Raji, Adaobi Solarin, Theophilus Umeizudike, Ernestina Eduful, Bola Fatima Olokode, Nanna Ripiye, Tolulope Oluwadare, Rotimi Braimoh, Chinwuba Ijoma, Amisu A. Mumuni, Yvette Cozier, Lisa Quintiliani, Fatiu Arogundade, Babatunde Salako, Rulan Parekh, Rasheed A. Gbadegesin, Josee Dupuis, Ifeoma Ulasi, Dwomoa Adu, Akinlolu Ojo, Sushrut Waikar, Diane Mitchell, Cheryl Anderson, Titilayo O. Ilori

**Affiliations:** 1 Division of Nephrology, Department of Medicine, Boston Medical Center, Boston University Chobanian & Avedisian School of Medicine, Boston, MA, United States; 2 Division of Public Health Sciences, Department of Epidemiology, Penn State College of Medicine, Hershey, PA, United States; 3 Department of Medicine and Therapeutics, University of Ghana Medical School, University of Ghana, Accra, Ghana; 4 Department of Medicine, College of Medicine, University of Lagos, Yaba, Nigeria; 5 Department of Medical Pharmacology and Therapeutics, Obafemi Awolowo University, Ile-Ife, Nigeria; 6 Department of Medicine, Obafemi Awolowo University, Ile-Ife, Nigeria; 7 Department of Medicine, College of Medicine, University of Ibadan, Ibadan, Nigeria; 8 Department of Pediatrics and Child Health, College of Medicine, Lagos State University, Ojo, Nigeria; 9 Department of Medicine, College of Medicine, University of Lagos, Lagos, Nigeria; 10 Department of Internal Medicine, University of Abuja, Abuja, Nigeria; 11 Department of Medicine, College of Medicine, University of Nigeria, Enugu, Nigeria; 12 Slone Epidemiology Center, Boston University Chobanian and Avedisian School of Medicine Boston, MA, United States; 13 Friedman School of Nutrition, Tufts Medical Center, Boston, MA, United States; 14 Department of Medicine, Women’s College Hospital, Hospital for Sick Children and University of Toronto, Toronto, ON, Canada; 15 Department of Pediatrics, Department of Medicine, Duke University School of Medicine, Durham, NC, United States; 16 Department of Epidemiology, Biostatistics and Occupational Health, McGill University, Montreal, QC, Canada; 17 Department of Medicine and Therapeutics, University of Ghana Medical School, College of Health Sciences, University of Ghana, Accra, Ghana; 18 Department of Medicine, Kansas University Medical Center, The University of Kansas, Lawrence, KS, United States; 19 Department of Nutrition, Texas A&M University, College Station, TX, United States; 20 Herbert Wertheim School of Public Health and Human Longevity Science, University of California San Diego, La Jolla, CA, United States

**Keywords:** beverage consumption patterns, blood pressure, chronic kidney disease, diabetes, hypertension, sub-Saharan Africa

## Abstract

**Introduction:**

Beverage intake is an important yet understudied contributor to blood pressure (BP) and proteinuria. This is particularly relevant in sub-Saharan Africa, where rapid shifts toward sugar-sweetened beverages (SSBs) and ultra-processed beverages, driven by affordability and aggressive marketing, are occurring alongside a high burden of Chronic Kidney Disease (CKD) within resource-limited health systems. Additionally, there are cultural differences within African populations that make beverage patterns in sub-Saharan Africa differ across populations and from Western cultures.

**Methods:**

We conducted a cross-sectional analysis of 494 participants in the Diet, CKD, and APOL1 (DCA) Study cohort in West Africa. We assessed beverage consumption from 24-h dietary recalls and patterns using principal component analysis. We analyzed associations of beverage patterns with systolic BP (SBP), diastolic BP (DBP), and proteinuria using univariate and multivariable linear mixed-effects regression models. We adjusted for covariates, such as clinical site (random effect), socio-demographic factors, and lifestyle factors.

**Results:**

We identified 4 unique beverage patterns: (i) SSB and Alcohol, (ii) Milk and Alcohol, (iii) SSB and Water without Alcohol, and (iv) Milk and Milk Products. No beverage patterns showed consistent association with SBP or DBP, and sensitivity analyses of individual beverages yielded null findings. In adjusted analyses, the Milk and Milk Products beverage pattern showed a positive association with SBP (Tertile 2 vs. 1: *β* = 5.61 mm Hg; 95% CI: 1.54–9.57) and a directionally consistent but not significant association in tertile 3 vs. tertile 1. An exploratory interaction suggested a stronger positive association of this pattern with SBP among individuals with diabetes.

**Conclusion:**

The Milk and Milk Products beverage pattern may be associated with higher SBP in adults with CKD in West Africa, with a potentially stronger association among those with diabetes. Given that no associations remained significant after false discovery rate correction, these findings should be interpreted cautiously. Future studies are needed to confirm these findings and clarify their long-term implications for kidney and cardiovascular health.

## Introduction

Beverages represent a substantial yet often overlooked component of dietary intake, contributing meaningfully to total caloric and nutrient consumption and accounting for approximately 20% of total calorie intake (1). In chronic kidney disease (CKD), beverage choices are particularly important because adequate water intake supports hydration and may reduce hormonal signals associated with kidney stress (2). Certain beverages, such as sugar-sweetened beverages (SSBs), may also raise blood pressure (BP) or increase exposure to additives such as phosphates and sodium, factors that accelerate CKD progression (3, 4). Despite their importance, beverage intake remains understudied in dietary pattern research, especially among CKD populations.

Low- and middle-income countries, including those in sub-Saharan Africa, are experiencing rapid shifts in beverage consumption driven by increased availability, affordability, and aggressive marketing of SSBs and ultra-processed beverages (5). These changes are occurring alongside a high burden of CKD and other non-communicable diseases in health systems that are often resource-limited (6). As beverage markets expand and dietary behaviors evolve, understanding beverage consumption patterns in sub-Saharan Africa has become increasingly important for identifying modifiable contributors to hypertension and CKD.

Although evidence exists linking individual beverages to CKD risk and related outcomes, findings remain inconsistent. For example, data from the 2007 Second Taiwanese Survey on Prevalences of Hypertension, Hyperglycemia, and Hyperlipidemia reported lower CKD risk among frequent and occasional drinkers compared with non-drinkers (7–9). In contrast, a Korean cohort found that, after multivariable adjustment, alcohol drinkers had a higher CKD risk, especially those with proteinuria or impaired kidney function (10). Large cohort studies from the UK Biobank and the Tehran Lipid and Glucose Study further suggest that high SSB intake increases incident CKD risk (11, 12), whereas high-fat milk and dairy may be protective of CKD (13). Additionally, tea, milk, and fruit beverages have been linked to elevated BP (14, 15), while polyphenol-rich juices and yogurt appear to lower BP (16, 17). Collectively, these findings highlight complex and beverage-specific relationships with CKD and its risk factors.

However, these studies provide limited insight into overall beverage patterns, defined as combinations of beverages typically consumed together that capture both type and frequency of intake (18). The few studies that do examine beverage profiles rely heavily on non-African populations, limiting generalizability to West Africa, where beverage markets, cultural practices, and CKD epidemiology differ substantially. For instance, in the Chronic Renal Insufficiency Cohort, a higher Healthy Beverage Score (characterized by higher intake of low-fat milk and coffee/tea, moderate alcohol, and lower intake of 100% fruit juice, artificially sweetened beverages, whole-fat milk, and SSBs) was associated with reduced CKD progression (19). Among Black Americans, a principal component-derived SSB pattern, characterized by higher intake of soda, sweetened fruit drinks, and water, was associated with greater odds of incident CKD (20).

Additionally, cultural heterogeneity across African populations shapes beverage consumption patterns in sub-Saharan Africa in ways that differ both within the region and relative to Western contexts (21). Many alcoholic and non-alcoholic beverages carry cultural and symbolic significance and are deeply embedded in social life and communal interactions, which can make established consumption patterns resistant to behavioral change (22). Traditional fermented cereal-based beverages, such as ogi, kunun, and mahewu, are widely consumed across age groups and are often perceived as foods rather than beverages. This cultural framing complicates the accurate reporting, classification, and regulation of these products in dietary assessments and public health interventions (23).

Taken together, several additional key gaps remain. Prior studies in West Africa have applied data-driven methods to derive overall dietary patterns that may include beverage-related components (24–26); however, beverage-specific patterning has rarely been examined, particularly in relation to kidney-relevant outcomes among adults with CKD. This gap is especially relevant in West Africa, where CKD burden is disproportionately high (19.8%) compared with other African populations (27). While some West African studies have evaluated dietary or beverage-related patterns in relation to BP (28), no studies to our knowledge have jointly examined beverage consumption patterns in relation to both BP and proteinuria, two critical markers of cardiovascular and kidney disease progression (29, 30). Moreover, beverage consumption habits in West Africa (e.g., traditional herbal preparations) differ substantially from those in Western populations (31), and many existing studies are limited by small sample sizes or narrow outcome assessment, constraining their ability to inform kidney-specific dietary guidance in this region (32).

This critical gap limits the development of culturally relevant dietary guidance and risk-reduction strategies. To address this deficit, we aim to (1) define patterns of beverage consumption among West Africans and (2) evaluate the association of beverage patterns with BP and proteinuria in this CKD cohort. We leveraged data from the Diet, CKD, and APOL1 (DCA) Study, a cohort of adults with CKD in Ghana and Nigeria (33). Our hypothesis is that unhealthy beverage patterns such as SSB consumption are associated with higher SBP and DBP, as well as elevated proteinuria. Identifying beverages that may have protective or adverse effects on kidney health in this population could provide valuable insights for dietary and clinical guidelines in CKD and hypertension prevention and management.

## Methods

### Study design and population

#### The DCA cohort study

We conducted a cross-sectional analysis of baseline data from the Diet, CKD, and APOL1 (DCA) Study (33), an ancillary study to the Human Heredity and Health in Africa (H3Africa) Kidney Disease Research Network (33, 34). The parent H3Africa Kidney Disease Study enrolled adults with CKD using standardized eligibility criteria and harmonized clinical protocols across sites (34). The goal of the parent study was to investigate genetic, environmental, and clinical determinants of kidney disease in sub-Saharan Africa (34).

The DCA study represents a convenience sample of individuals recruited from seven clinical sites who had not yet reached CKD outcomes (dialysis or renal replacement therapy) and were able to complete dietary assessments (33). The study recruited participants who had CKD and were older than 15 years of age. In addition, there were two groups of patients. In the first group/CKD group, the estimated glomerular filtration rate (eGFR) inclusion criterion was 20–59 mL/min per 1.73 m^2^. In the second group (the glomerulonephritis group), participants were required to have an eGFR >15 mL/min per 1.73 m^2^ and a biopsy-confirmed diagnosis of glomerulonephritis in the H3Africa cohort (33, 34). We performed kidney biopsies in individuals with an eGFR ≥15 mL/min per 1.73 m^2^ with substantial albuminuria, defined as an albumin-to-creatinine ratio (ACR) > 50 mg/mmol or albumin excretion exceeding 500 mg per 24 h. Biopsy specimens underwent standardized processing with light microscopy, immunofluorescence, and electron microscopy, and all samples were interpreted centrally at the University of Michigan and Massachusetts General Hospital pathology departments (35).

Exclusion criteria include: (i) individuals who are HIV positive, (ii) individuals who have received a kidney transplant, (iii) individuals who are institutionalized, (iv) individuals with New York Heart Association Class III or IV heart failure, (v) individuals with known cirrhosis, (vi) females who are pregnant or breastfeeding, (vii) individuals with polycystic kidney disease, (viii) individuals who have received a solid organ or bone marrow transplant, (ix) individuals with chronic obstructive uropathy, (x) individuals with adult-onset monogenic kidney disease, (xi) individuals with mitochondrial disease, (xii) individuals with maturity-onset diabetes of the young, and (xiii) individuals with kidney failure. Enrollment of study subjects started in February 2021 and continued for 2 years. Annual and six-month follow-up visits began in February 2022.

The DCA study was approved by the Institutional Review Board of Boston Medical Center (H-40137) and all the participating sites (33), and the full protocol for the study has been described previously (33).

### Beverage pattern assessment

The DCA study assessed dietary intake at baseline using up to two non-consecutive 24-h dietary recalls administered by trained dietitians at clinical sites (36). Participants scheduled their study visits at their convenience; however, each recall captured all foods and beverages consumed during the day immediately preceding the study visit, and participants did not select a preferred day for dietary reporting. Participants were first instructed to record their dietary intake from the previous day on a standardized form provided by the study team (33). During the study visit, trained study coordinators conducted in-depth, interviewer-administered recalls using structured probing questions to clarify portion sizes, preparation methods, and to ensure complete capture of all food and beverage items consumed. This procedure is described in further detail elsewhere (33). Each beverage reported was linked to the West African Food Composition Table to obtain gram weights and nutrient content (37). Free text entries were reviewed, and local dietitians supplemented the food list with region-specific items. Nutrient profiles for these items were determined through consensus between Africa-based and U. S.-based dietitians at Penn State University to ensure cultural accuracy. Analysts reviewed all beverage entries for completeness and plausibility before analysis, and items identified as foods rather than beverages were excluded.

We developed a beverage classification system specifically for this study, and registered dietitians at participating sites reviewed the classification for accuracy and validity. We assigned each beverage code to one of eight mutually exclusive beverage groups: alcohol (e.g., beer, palm wine, whiskey, Irish cream liqueur), coffee, dairy milk and its products, juice (e.g., sweetened and unsweetened), plant milk/drink (e.g., soybean milk, kunu, a plant-based drink from tigernuts), tea (e.g., chocolate drinks including breakfast hot chocolate and tea infusions), soda (e.g., carbonated drinks, energy drinks, ginger ale), and other beverages (e.g., water and coconut water). The classification of individual beverage items into beverage groups is provided in Supplementary Table 1. In this population, most tea and coffee consumed included added sugar. For each recall day, we summed the gram intake of all beverages within each group. We then averaged daily group intakes (g/day) across available recall days to estimate each participant’s usual beverage intake. We coded non-consumption of a beverage group as zero. Mean daily gram intakes for each beverage group were used as inputs for principal components analysis (PCA). Dietary recalls were not collected on festival days or holidays due to known variation in beverage consumption.

Before conducting PCA, we standardized each beverage group to a mean of 0 and a standard deviation of 1. We then applied PCA to the correlation matrix to identify underlying beverage consumption patterns, with factor loadings quantifying the correlation between each beverage group and each principal component. We calculated participant-specific component scores as weighted linear combinations of beverage group intakes, reflecting the degree of adherence to each pattern. We retained components with interpretable structures and used beverage groups with absolute factor loadings ≥0.20 to label each pattern. We treated scores for the first four components as continuous exposures and additionally categorized them into tertiles for regression analyses, using the lowest tertile as the reference group.

### Study outcome

The primary outcomes of this study were systolic BP (SBP) and diastolic BP (DBP). Study coordinators measured three sitting BPs during the study visit using the Omron HEM-907XL pro BP monitor, and the average SBP and DBP were used as the outcome. BP was also measured on the day the urine specimen was returned to the clinical centers. The secondary outcome was proteinuria, assessed from 24-h urine protein collections. Urine samples were processed in laboratories in Nigeria and Ghana, where protein quantification was performed.

### Covariate measurements

Study coordinators collected data on age, sex, and clinical site using case report forms in the DCA study. Height and weight were measured during study visits, and body mass index (BMI) was calculated for analysis. Additional data on income (high vs. low/medium), educational status (higher vs. secondary/less), alcohol use, smoking status, hypertension, and diabetes were obtained from enrollment data collected in the parent H3Africa Kidney Disease Study. Data on hypertension, antihypertensive medication use, and diabetes were self-reported. Antihypertensive medication use was defined as current use of diuretics, angiotensin-converting enzyme (ACE) inhibitors, angiotensin receptor blockers (ARBs), or other prescription medications used to lower BP. The eGFR (mL/min per 1.73 m^2^) was calculated using the 2009 Chronic Kidney Disease Epidemiology Collaboration equation without adjustment for Black race (38–40). Total energy intake was estimated using 24-h dietary recalls and included as a covariate in sensitivity analyses.

### Statistical analysis

We used descriptive statistics to summarize baseline characteristics of the study population, stratified by tertiles of each beverage pattern. Continuous variables were summarized using means and standard deviations or medians and interquartile ranges, as appropriate. Differences across tertiles were assessed using one-way analysis of variance or Kruskal–Wallis tests for continuous variables and chi-square tests for categorical variables.

We fitted two linear mixed-effects regression models to estimate the effects of beverage consumption patterns on SBP, DBP, and proteinuria, presenting the estimates with 95% confidence intervals (CIs). Mixed models were selected because they appropriately account for clustering within clinical sites and accommodate the correlation structure arising from repeated measures within participants. Beverage pattern scores were modeled as continuous variables and as tertiles. Model 1, the univariate analysis, only included the exposure of interest (beverage patterns). Beverage patterns were included in each model as either categorical (tertiles) or continuous variables. In Model 2, we adjusted for age, sex, education, income, baseline eGFR, smoking, diabetes, BMI, and total energy intake; hypertension status was additionally adjusted for in the proteinuria model, and proteinuria was adjusted for in the hypertension models. We also adjusted for total energy intake to mitigate extraneous variation potentially introduced by the food frequency questionnaire (41). Mixed-effects models were evaluated using standard diagnostic procedures. The assumptions of normality, homoscedasticity, and linearity were assessed through residual diagnostics, including Q–Q plots and residuals versus fitted values plots. No violations of these assumptions were detected, indicating that the model was appropriately specified.

We calculated the p for trend across tertiles, with statistical significance at *p* < 0.05. We performed sensitivity analyses in the association of Beverage Patterns with SBP, DBP, and proteinuria, adjusting for antihypertensive medications in the fully adjusted models (Supplementary Table 2) (Model 2 + BP medications). In exploratory analyses, we examined potential effect modification of kidney function (eGFR), age, sex, BMI, hypertension, and diabetes on the association between beverage patterns with SBP using tests of interaction and stratified analyses (Table 1). Because these analyses were exploratory in nature, *p*-values for interaction were not adjusted for multiple comparisons (42). We applied false discovery rate (FDR) correction using the Benjamini–Hochberg procedure to the primary beverage pattern–outcome associations (12 tests per model) to control for multiple testing. We report adjusted *p* values (*q*-values) alongside unadjusted p-values and define statistical significance after correction as *q* < 0.05. We conducted a complete case analysis excluding participants with no beverage consumption, and we assumed non-consumption of beverages to be zero.

**Table 1:** Association between Milk and Milk Products beverage pattern and systolic blood pressure by kidney function, age, sex, obesity status, and diabetes status for participants in the Diet, CKD, and APOL1 (DCA) study (2021–2023).

Subgroup	Systolic blood pressure					
	Categorical [Estimate (95% CI)]			*P* for interaction	Continuous	*P* for interaction
	Tertile 1	Tertile 2	Tertile 3		Estimate (95% CI)	
eGFR 60- < 90 mL/min per 1.73 m^2^	Ref	**6.91 (1.50 to 12.19)**	3.35 (−2.37 to 8.81)	0.82	0.38 (−2.19 to 2.86)	0.89
eGFR ≥ 90 mL/min per 1.73 m^2^	Ref	2.43 (−3.47 to 8.18)	0.12 (−5.58 to 5.72)		0.03 (−1.79 to 1.84)	
Age <50 years	Ref	3.12 (−2.23 to 8.38)	0.45 (−4.44 to 5.26)	0.42	−0.61 (−2.47 to 1.27)	0.66
Age ≥ 50 years	Ref	**6.59 (0.40 to 12.40)**	5.70 (−1.70 to 12.15)		0.14 (−2.87 to 2.76)	
Male	Ref	5.74 (−0.26 to 11.49)	3.34 (−2.79 to 9.12)	0.71	−0.40 (−2.59 to 1.73)	0.71
Female	Ref	4.89 (−0.65 to 10.30)	2.03 (−3.57 to 7.49)		0.52 (−1.92 to 2.91)	
BMI < 25 kg/m^2^	Ref	5.65 (−0.50 to 11.65)	4.80 (−1.41 to 10.85)	0.65	−0.79 (−3.18 to 1.59)	0.23
BMI 25- < 30 kg/m^2^	Ref	5.14 (−2.98 to 12.12)	−1.65 (−9.28 to 5.17)		−0.72 (−3.46 to 2.05)	
BMI ≥ 30 kg/m^2^	Ref	4.29 (−2.43 to 11.33)	1.25 (−5.93 to 8.44)		2.93 (−0.10 to 5.96)	
Diabetes	Ref	**14.70 (5.08 to 22.79)**	**15.58 (3.91 to 25.19)**	**0.04**	4.26 (−1.33 to 9.17)	0.12
No diabetes	Ref	2.96 (−1.53 to 7.37)	0.50 (−3.91 to 4.80)		−0.27 (−1.92 to 1.37)	
Hypertension	Ref	4.50 (−0.61 to 9.52)	1.03 (−4.38 to 6.31)	0.87	−0.19 (−2.67 to 2.21)	0.88
No hypertension	Ref	0.63 (−3.95 to 5.09)	1.96 (−2.15 to 6.01)		−0.38 (−1.95 to 1.24)	

Multivariable mixed-linear regression models with clinical site as random effect. Age, gender, education, income, proteinuria, estimated glomerular filtration rate (eGFR), smoking, diabetes, BMI, and total energy (per 1,000 KCAL). The participants received a score for each pattern based on their consumption and the PC scores were divided into 3 equal groups to facilitate comparisons. Tertile 3 represents the highest adherence to a specific beverage pattern, tertile 2 moderate, and tertile 1 the lowest. Age, proteinuria, eGFR, BMI, and total energy were *z*-score normalized to improve the convergence of the regression algorithm.

Bold values indicate statistically significant associations at *p* < 0.05.

We also conducted sensitivity analyses for SBP to evaluate the associations of individual beverage groups independent of beverage pattern derivation, in order to assess whether any observed pattern-level associations were robust to alternative exposure definitions (Supplementary Table 3). For each beverage group (alcohol, dairy milk and its products, soda, tea, coffee, juice, plant milk/drink, and other beverages), we fitted two linear mixed-effects regression models with clinical site included as a random effect. Model 1 included the beverage group of interest and the random effect for clinical site. Model 2 was fully adjusted and included age, sex, education, income, proteinuria, baseline eGFR, smoking status, diabetes status, BMI, and total energy intake. All beverage variables were standardized (z-scored) prior to analysis to allow comparison of effect sizes across beverage groups. Missing beverage consumption data were coded as zero, reflecting non-consumption during the dietary recall period, consistent with standard 24-h dietary recall analytic approaches.

All analyses were performed using RStudio version 4.2.3 and later.

## Results

### Beverage pattern identification

Using PCA, we identified four primary beverage consumption patterns among participants (Table 2). *Beverage Pattern 1*—SSB and Alcohol—was characterized by high intake of soda and alcohol, coupled with low consumption of dairy milk, milk products, and tea. *Beverage Pattern 2*—Milk and Alcohol—was characterized by high consumption of alcohol, dairy milk, milk products, and plant-based drinks, with low intake of coffee, juice, and soda. *Beverage Pattern 3*—SSB and Water Without Alcohol—was characterized by high consumption of soda and other beverages (such as tap and coconut water) and low intake of alcohol, coffee, juice, and plant-based drinks. *Beverage Pattern 4*—Milk and Milk Products—was characterized by high consumption of dairy milk and its products, with low consumption of other beverages, tea, and plant-based drinks (Table 2).

**Table 2:** Factor loadings for beverages in the individual beverage patterns in the Diet, CKD, and APOL1 (DCA) study.

Beverage type	PC1	PC2	PC3	PC4
Alcohol beverage	0.21	0.57	−0.38	–
Coffee	–	−0.43	−0.44	–
Dairy milk and its products	−0.32	0.23	–	0.65
Juice	–	−0.5	−0.52	–
Other beverages	–	–	0.26	−0.57
Plant milk/drink	–	0.29	−0.28	−0.34
Soda	0.54	−0.27	0.47	–
Tea	−0.68	–	–	−0.3
Variance explained (%)	15.40	14.48	13.92	12.21

PC- principal component.

Each beverage consumption pattern was defined by the correlation of the PCs with the beverage consumed (± 0.20). Greater than or equal to 0.20 denotes high intake, while less than or equal to −0.20 denotes low intake.

PC1- SSB and Alcohol beverage pattern, PC2- Milk and Alcohol beverage pattern, PC3- SSB and Water without Alcohol beverage pattern, and PC4- Milk and Milk Products beverage pattern.

Together, these four beverage consumption patterns explained 56.0% of the total variance in beverage intake across individuals. Variance refers to the proportion of overall differences in beverage consumption across individual that is captured by each pattern. Specifically, the patterns accounted for 15.4, 14.5, 13.9, and 12.2% of the variance, respectively. We defined each beverage consumption pattern using factor loadings derived from PCA, which quantify the correlation between each beverage intake variable and the corresponding principal component. Beverages with factor loadings > 0.20 were considered to contribute positively and represent relatively higher intake within a pattern, whereas beverages with loadings < −0.20 were considered to contribute negatively and represent relatively lower intake.

### Baseline characteristics

Supplementary Table 2 summarizes the baseline characteristics of 494 of the 758 participants in the DCA cohort with complete beverage patterns. The mean age of participants in the cohort was 49 years (SD = 18). The median (IQR) eGFR was 67 (38.78, 98) mL/min per 1.73 m^2^ and 24-h protein was 0.34 (0.16, 1.12) mg. The population was 48% female, 70% had ≤ secondary school education, 5.8% were smokers, and 43% reported alcohol consumption (Supplementary Table 4). Baseline characteristics across Beverage Patterns are reported in Table 3.

**Table 3:** Baseline characteristics of participants across tertiles of beverage patterns in the Diet, CKD, and APOL1 (DCA) study (2021–2023).

	SSB and Alcohol beverage pattern			
Characteristics	Tertile 1 (*N* = 163^*^)	Tertile 2 (*N* = 166^*^)	Tertile 3 (*N* = 165^*^)	*p*-value^**^
Age, years	53 (18)	50 (18)	43 (16)	<0.001
BMI, kg/m^2^	26.7 (5.4)	26.1 (5.4)	26.2 (6.2)	0.243
Female	89 (55%)	84 (51%)	64 (39%)	0.012
Education^+^				0.239
Higher	121 (74%)	109 (66%)	115 (70%)	
Secondary/less	42 (26%)	57 (34%)	49 (30%)	
Income				0.032
High	8 (4.9%)	7 (4.2%)	9 (5.5%)	
Low/Medium	73 (45%)	94 (57%)	101 (61%)	
Smoking^+^	4 (2.5%)	11 (6.8%)	13 (8.0%)	0.081
Antihypertensive medication	117 (72%)	109 (66%)	125 (76%)	0.125
Drinking^+^	69 (43%)	65 (40%)	73 (45%)	0.666
SBP, mm Hg	133 (21)	131 (21)	127 (20)	0.046
Diabetes	52 (32%)	39 (23%)	11 (6.7%)	<0.001
eGFR, CKD-EPI 2009, mL/min per 1.73 m^2^	59 (35.8, 93)	74.2 (33.6, 99.1)	71.7 (46, 104)	0.068
Proteinuria, mg	0.25 (0.14, 0.93)	0.36 (0.16, 1.05)	0.38 (0.17, 1.38)	0.109

	Milk and Alcohol beverage pattern			
	Tertile 1 (*N* = 163^*^)	Tertile 2 (*N* = 164^*^)	Tertile 3 (*N* = 164^*^)	*p*-value^**^
Age, years	45 (18)	53 (17)	48 (18)	<0.001
BMI, kg/m^2^	26.3 (6.0)	26.6 (5.5)	26.1 (5.6)	0.579
Female	68 (42)	85 (52)	84 (51)	0.122
Education^+^				0.204
Higher	106 (65)	117 (71)	121 (74)	
Secondary/less	57 (35)	47 (29)	43 (26)	
Income				0.743
High	5 (3.1)	9 (5.5)	10 (6.1)	
Low/medium	90 (55)	90 (55)	86 (52)	
Smoking^+^	9 (5.6)	8 (5.1)	11 (6.8)	0.793
Antihypertensive medication	122 (75)	118 (72)	109 (66)	0.236
Drinking^+^	72 (45)	72 (46)	63 (39)	0.420
SBP, mm Hg	127 (21)	134 (22)	130 (19)	0.015
Diabetes	22 (13)	48 (29)	32 (20)	0.002
eGFR, CKD-EPI 2009, mL/min per 1.73 m^2^	72 (43.8, 106)	56.5 (24.5)	74.8 (43, 99)	0.002
Proteinuria, mg	0.32 (0.16, 1.00)	0.36 (0.17, 1.13)	0.33 (0.15, 1.05)	0.766

	Sugar-Sweetened and Water without Alcohol beverage pattern			
	Tertile 1 (*N* = 163^*^)	Tertile 2 (*N* = 164^*^)	Tertile 3 (*N* = 165^*^)	*p*-value^**^
Age, years	47 (18)	52 (18)	46 (18)	0.007
BMI, kg/m^2^	26.8 (5.6)	26.0 (5.5)	26.3 (6.0)	0.488
Female	67 (41)	94 (57)	76 (46)	0.011
Education^+^				0.979
Higher	114 (70)	114 (70)	116 (70)	
Secondary/less	48 (29)	50 (30)	49 (30)	
Income				0.742
High	10 (6.1)	9 (5.5)	5 (3.0)	
Low/medium	88 (54)	89 (54)	90 (55)	
Smoking^+^	10 (6.2)	6 (3.8)	12 (7.4)	0.380
Antihypertensive medication	115 (71)	113 (69)	122 (74)	0.589
Drinking^+^	72 (45)	65 (41)	70 (43)	0.810
SBP, mm Hg	130 (19)	133 (21)	128 (22)	0.126
Diabetes	24 (15)	47 (29)	31 (19)	0.006
eGFR, CKD-EPI 2009, mL/min per 1.73 m^2^	67 (39.5, 98.2)	66 (28.9, 96.5)	69.5 (44, 102)	0.328
Proteinuria, mg	0.36 (0.15, 1.30)	0.35 (0.17, 0.98)	0.31 (0.16, 1.16)	0.952

	Milk and Milk Products beverage pattern			
	Tertile 1 (*N* = 164^*^)	Tertile 2 (*N* = 164^*^)	Tertile 3 (*N* = 160^*^)	*p*-value^**^
Age, years	51 (18)	51 (17)	44 (18)	<0.001
BMI, kg/m^2^	26.7 (5.3)	26.4 (5.5)	25.8 (6.1)	0.082
Female	72 (44%)	85 (52%)	78 (49%)	0.350
Education^+^				0.651
Higher	118 (72%)	111 (68%)	112 (70%)	
Secondary/less	45 (27%)	53 (32%)	48 (30%)	
Income				0.398
High	11 (6.7%)	6 (3.7%)	6 (3.8%)	
Low/Medium	88 (54%)	95 (58%)	81 (51%)	
Smoking^+^	7 (4.3%)	12 (7.5%)	9 (5.7%)	0.473
Antihypertensive medication	126 (77%)	116 (71%)	105 (66%)	0.084
Drinking^+^	75 (47%)	64 (40%)	67 (43%)	0.514
SBP, mm Hg	131 (22)	132 (21)	128 (20)	0.197
Diabetes	38 (23%)	37 (23%)	26 (16%)	0.236
eGFR, CKD-EPI 2009, mL/min per 1.73 m^2^	56.5 (31, 94.8)	73.1 (38, 96.6)	75 (47.1, 111)	0.007
Proteinuria, mg	0.31 (0.14, 1.19)	0.34 (0.16, 1.11)	0.37 (0.17, 1.02)	0.899

eGFR, CKD-EPI 2009- estimated glomerular filtration rate, chronic kidney disease epidemiology collaboration 2009 not corrected for race. SBP- systolic blood pressure ^*^Mean (SD); *n* (%); Median (Q1, Q3). ^**^ Kruskal-Wallis rank sum test; Pearson’s Chi-squared test; Fisher’s exact test ^+^ 1 missing for education; 9–10 missing for smoking; 10–11 missing for drinking; 201–202 do not wish to answer for income.

### Association between beverage patterns with SBP, DBP, and proteinuria

In unadjusted analyses, higher tertiles of the SSB and Alcohol Beverage Pattern showed inverse associations with SBP (Tertile 2 *β* = −0.55 mm Hg, 95% CI –5.06 to 3.92; Tertile 3 *β* = −4.49 mm Hg, 95% CI –9.03 to −0.04; p for trend = 0.05; Table 4). No consistent associations were observed between other beverage patterns and SBP, DBP, or proteinuria in unadjusted analyses (Table 4).

**Table 4:** Association of beverage patterns with systolic and diastolic blood pressure, and proteinuria for participants in the Diet, CKD, and APOL1 (DCA) Study (2021–2023).

Exposure level	SBP^*^		DBP*		Proteinuria^+^	
	Model 1 [Estimate (95% CI)]	Model 2 [Estimate (95% CI)]	Model 1 [Estimate (95% CI)]	Model 2 [Estimate (95% CI)]	Model 1 [Estimate (95% CI)]	Model 2 [Estimate (95% CI)]
Sugar-Sweetened Beverage and Alcohol beverage pattern						
Tertile 1	Ref	Ref	Ref	Ref	Ref	Ref
Tertile 2	−0.55 (−5.06, 3.92)	1.15 (−2.95, 5.18)	0.31 (−2.68, 3.22)	−0.04 (−3.01, 2.88)	0.12 (−0.16, 0.42)	0.03 (−0.25, 0.32)
Tertile 3	**−4.49 (−9.03, −0.04)**	−1.58 (−5.75, 2.48)	1.84 (−1.26, 4.74)	0.56 (−2.49, 3.49)	0.24 (−0.04, 0.54)	0.01 (−0.27, 0.30)
*P* for trend	**0.05**	0.47	0.22	0.72	0.10	0.97
q (FDR)	0.41	0.80	0.44	0.96	0.41	0.97
Continuous	−0.74 (−2.40, 0.90)	0.30 (−1.20, 1.77)	0.93 (−0.19, 1.99)	0.65 (−0.45, 1.71)	0.08 (−0.03, 0.19)	−0.00 (−0.10, 0.11)
Milk and Alcohol beverage pattern						
Tertile 1	Ref	Ref	Ref	Ref	Ref	Ref
Tertile 2	**6.55 (2.15, 10.95)**	3.07 (−0.96, 7.17)	1.57 (−1.31, 4.53)	0.82 (−2.09, 3.82)	0.12 (−0.17, 0.40)	0.11 (−0.18, 0.38)
Tertile 3	3.11 (−1.38, 7.55)	2.48 (−1.39, 6.32)	2.15 (−0.75, 5.12)	2.38 (−0.38, 5.22)	0.03 (−0.26, 0.32)	0.11 (−0.16, 0.38)
*P* for trend	0.17	0.22	0.15	0.10	0.82	0.44
q (FDR)	0.41	0.80	0.41	0.80	0.90	0.80
Continuous	1.24 (−0.45, 2.91)	0.79 (−0.73, 2.29)	0.58 (−0.52, 1.68)	0.77 (−0.31, 1.86)	−0.02 (−0.13, 0.09)	0.02 (−0.09, 0.12)
Sugar-Sweetened Beverage and Water without Alcohol beverage pattern						
Tertile 1	Ref	Ref	Ref	Ref	Ref	Ref
Tertile 2	2.89 (−1.54, 7.29)	1.54 (−2.37, 5.46)	−1.02 (−3.91, 1.90)	−0.52 (−3.33, 2.34)	−0.02 (−0.31, 0.26)	0.02 (−0.25, 0.29)
Tertile 3	−1.02 (−5.45, 3.36)	−1.24 (−5.11, 2.58)	−2.02 (−4.94, 0.86)	−1.56 (−4.37, 1.21)	−0.01 (−0.30, 0.27)	0.03 (−0.24, 0.30)
*P* for trend	0.65	0.53	0.17	0.28	0.95	0.80
q (FDR)	0.89	0.80	0.41	0.80	0.95	0.96
Continuous	−0.09 (−1.80, 1.61)	0.61 (−0.89, 2.09)	−0.11 (−1.23, 1.01)	0.11 (−0.98, 1.18)	−0.02 (−0.13, 0.09)	−0.04 (−0.15, 0.06)
Milk and Milk Products beverage pattern						
Tertile 1	Ref	Ref	Ref	Ref	Ref	Ref
Tertile 2	2.91 (−1.71, 7.37)	**5.61 (1.54, 9.57)**	1.39 (−1.59, 4.31)	1.95 (−0.94, 4.87)	0.05 (−0.25, 0.34)	0.04 (−0.24, 0.32)
Tertile 3	−0.78 (−5.47, 3.74)	3.03 (−1.09, 7.00)	0.52 (−2.47, 3.49)	0.94 (−1.96, 3.87)	0.11 (−0.19, 0.41)	0.01 (−0.26, 0.29)
*P* for trend	0.74	0.15	0.73	0.53	0.46	0.92
q (FDR)	0.89	0.80	0.89	0.80	0.79	0.97
Continuous	−0.54 (−2.42, 1.30)	0.27 (−1.35, 1.86)	0.43 (−0.76, 1.63)	0.19 (−0.95, 1.40)	0.05 (−0.08, 0.17)	−0.01 (−0.13, 0.09)

Model 1: included beverage pattern and the random effect (clinical site). Model 2: Adjusted for age, sex, education, income, ^*^proteinuria, ^+^hypertension, estimated Glomerular Filtration Rate (eGFR), smoking, diabetes, BMI, and total energy (per 1,000 KCAL). The participants received a score for each pattern based on their consumption and the PC scores were divided into 3 equal groups to facilitate comparisons. Tertile 3 represents the highest adherence to a specific beverage pattern, tertile 2 moderate, and tertile 1 the lowest. q (FDR) = FDR-adjusted p-values (Benjamini–Hochberg; 12 tests per model).

Bold values indicate statistically significant associations at p < 0.05.

After adjusting for covariates, compared to tertile 1 (reference), tertile 2 of the Milk and Milk Products beverage pattern showed a positive association with higher SBP, while tertile 3 remained directionally similar but a non-significant association (Tertile 2 *β* = 5.61 mm Hg, 95% CI 1.54–9.57; Tertile 3 *β* = 3.03 mm Hg, 95% CI –1.09 to 7.00; p-trend = 0.15). After controlling for the FDR across 12 tests within each model, *q*-values were also > 0.05, confirming that none of the observed trends met criteria for statistical significance. The positive direction of the Milk and Milk Products–SBP association remained consistent.

### Effect modification

The association of Milk and Milk Products with SBP varied depending on diabetes status (*p* for interactio*n* = 0.04, Table 1). Among individuals with diabetes, higher tertiles of this pattern were associated with higher SBP [tertile 2 vs. tertile 1: *β* = 14.7, 95% CI: 5–22.3; tertile 3 vs. tertile 1: *β* = 15.6, 95% CI: 3.9–25.2].

### Sensitivity analyses

To evaluate the robustness of our findings, we performed sensitivity analyses additionally adjusting for use of BP–lowering medications. The associations between beverage patterns and SBP, DBP, and proteinuria were materially unchanged after this adjustment (Supplementary Table 2).

In sensitivity analyses examining individual beverage groups and SBP, we fitted two sequential linear mixed-effects models (Supplementary Table 3). Across both models, no individual beverage groups, including alcohol, dairy milk and its products, soda, tea, coffee, juice, plant milk/drink, or other beverages, were significantly associated with SBP.

## Discussion

This study examined the associations of beverage consumption patterns with SBP, DBP, and proteinuria among CKD participants in Ghana and Nigeria. We identified four beverage patterns in this West African CKD cohort: the SSB and Alcohol Beverage Pattern, the Milk and Alcohol beverage pattern, the SSB and Water Without Alcohol beverage pattern, and the Milk and Milk Products beverage pattern. We observed no associations of beverage patterns with SBP or DBP. Although no significant trend was observed, the Milk and Milk Products beverage pattern was associated with higher SBP in the second tertile only, with effect estimates remaining directionally positive across increasing intake levels. Our exploratory interaction analyses showed that among those with diabetes, adherence to the Milk and Milk Products beverage pattern appeared to be associated with higher SBP (p for interactio*n* = 0.04, Table 1).

Prior studies conducted in Western populations have generally reported no significant increase in BP associated with dairy intake (43). Consistent with this literature, we observed overall null associations between adherence to the Milk and Milk Products beverage pattern and SBP in the present study. Although a statistically significant association was observed in a single tertile, the absence of a significant trend across tertiles suggests that this finding should be interpreted cautiously and does not support a robust positive association.

Differences in the types of dairy products consumed across regions may nonetheless be relevant for contextual interpretation. In West Africa, dairy intake often includes processed forms such as powdered milk, evaporated or tinned milk, and flavored yogurts, including imported products that may contain added sugars (44). These products differ from fresh cow’s milk, which predominates in Western dietary patterns, and may vary in added sugar content as well as fat and sodium composition (37). Processing may additionally alter bioactive peptides proposed to contribute to the BP–lowering effects of dairy products (45). Excess dietary sugar intake has been implicated in elevated BP through mechanisms related to insulin resistance, sodium retention, inflammation, and oxidative stress, which are also relevant to kidney injury (46–50). However, the contribution of added sugars within dairy products could not be directly evaluated in this study and warrants further investigation in longitudinal research.

We found no association of the SSB and Alcohol beverage pattern with SBP. This finding differs from previous studies reporting positive associations of SSB intake with incident hypertension and CKD (51). Sensitivity analyses of individual beverage groups did not reveal any statistically significant associations with SBP after multivariable adjustment. One possible explanation for these null findings is reverse causality, whereby individuals with advanced CKD may have already reduced their intake of soda or other SSBs following dietary counseling. This interpretation is supported by lower SBP levels and a lower prevalence of diabetes observed across higher tertiles of the SSB and Alcohol beverage pattern (Table 3).

Differences from studies reporting positive associations between SSBs and hypertension or CKD may reflect the structure of PCA-derived patterns. In this cohort, SSBs clustered with alcohol consumption, creating a mixed pattern that captures co-consumption rather than isolated beverage effects. Alcohol intake has been shown to exhibit heterogeneous associations with SBP depending on drinking level, beverage type, metabolic health, and lifestyle factors (52–54). Accordingly, null associations in this pattern may reflect the combination of beverages with opposing physiological effects rather than a lack of association for any single beverage. More broadly, PCA-derived patterns can yield attenuated or null associations when exposures with divergent health effects cluster together (55).

The absence of significant findings for DBP across all patterns aligns with previous observations that DBP exhibits less responsiveness to dietary interventions compared to SBP (56).

This study provides several strengths. To our knowledge, it is one of the first to characterize beverage consumption patterns within a CKD population in sub-Saharan Africa, offering important insight into dietary behaviors in a region where such data remain limited. By employing PCA, we were able to empirically derive beverage patterns that reflect real-world consumption behaviors, enhancing ecological validity. Dietary data were collected through detailed, interviewer-administered 24-h recalls, which improves accuracy relative to self-administered recalls. Standardized BP measurements and harmonized data collection across clinical sites strengthened internal consistency. Models were adjusted for a wide range of sociodemographic and clinical covariates, improving internal validity. Furthermore, inclusion of participants from multiple sites in Nigeria and a site in Ghana represents an additional strength of this study, enhancing representation across two West African countries. Finally, the leveraging of a well-characterized CKD cohort provides a robust framework for examining dietary correlates of kidney health in this population.

Several limitations, however, must be acknowledged. Although we accounted for multiple testing using an FDR correction, none of the beverage pattern–outcome associations met statistical significance, indicating that the observed patterns should be interpreted with caution and viewed as hypothesis-generating to guide future studies. Reliance on self-reported dietary data may introduce recall bias, particularly for alcohol and SSB intake. Additionally, we did not differentiate weekend from weekday food and beverage consumption, which is a potential limitation because intake may vary across the week. However, coordinators instructed participants to select a day they felt was most representative of their usual eating patterns. Residual confounding cannot be ruled out, as unmeasured factors such as physical activity, stress, genetics, and broader dietary patterns may have influenced our findings. The relatively small sample size may have constrained our ability to detect significant associations for certain beverage patterns. We also examined several potential effect modifiers, and although an interaction with diabetes was observed, these tests were exploratory and unadjusted for multiple comparisons (36, 42). Although PCA captures shared beverage consumption patterns, it cannot fully isolate the effects of individual beverages when they cluster together; our complementary mutually adjusted models address this in part, but beverage-specific estimates remain exploratory. Despite these limitations, the study offers important preliminary insights into beverage intake among West Africans with CKD.

We cannot infer causality in our analyses because of the cross-sectional design of the study. However, our study is important because it is a detailed characterization of beverage consumption patterns in a low-resource setting with a paucity of dietary data, especially beverage data. Our data provide detailed, recall-based information on habitual beverage intake across multiple clinical sites in Ghana and Nigeria, enabling data-driven identification of beverage patterns within a well-characterized CKD cohort (57). Our findings also highlight the importance of considering multiple hypothesis testing in pattern-based dietary analyses. Application of FDR correction helped ensure that observed associations were robust to potential inflation of Type I error. Future work should incorporate longitudinal study designs to clarify causality and evaluate whether changes in beverage intake influence SBP or kidney outcomes. Larger samples with repeated dietary assessments will be valuable for refining beverage patterns and understanding their health implications. Given the high burden of metabolic disease in the region, it will also be important to determine whether individuals with diabetes or other metabolic conditions exhibit heightened sensitivity to certain beverage patterns. Integrating genetic factors such as APOL1 risk variants may further elucidate population-specific susceptibility to hypertension and CKD.

## Conclusion

This study found limited associations between beverage consumption patterns and SBP, DBP, and proteinuria in a West African CKD cohort. Overall, no beverage patterns or individual beverages were associated with SBP or DBP. Modest associations observed for the Milk and Milk Products beverage pattern, along with exploratory indications of potential effect modification by diabetes, warrant further investigation and should be confirmed in larger, longitudinal studies. Importantly, milk and dairy-derived products consumed in this region are nutritionally heterogeneous and often include processed forms such as powdered, condensed, or sweetened dairy, which may differ from fresh milk in their sugar, fat, and sodium content. Collectively, these findings underscore the need for longitudinal research to evaluate the long-term health effects of beverage consumption patterns and to inform the development of culturally relevant dietary guidance for West African populations.

### Practical application

The observed positive association of the Milk and Milk Products beverage pattern with SBP, although exploratory and not statistically significant after correction for multiple testing, was directionally consistent with higher SBP values. Larger studies are needed to more precisely evaluate the association between beverage patterns and BP. Future work using longitudinal data will be important for determining whether beverage consumption patterns are associated with changes in BP or CKD progression over time.

## Data Availability

The raw data supporting the conclusions of this article will be made available by the authors, without undue reservation.
