# Supplementary material for: Beverage patterns, blood pressure, and proteinuria among West Africans with chronic kidney disease: a cross-sectional analysis of the diet, CKD, and apolipoprotein L1 study
Source: Front Nutr. 2026 Feb 6;13:1724375. doi: 10.3389/fnut.2026.1724375 (PMC12920209; doi:10.3389/fnut.2026.1724375)
Supplement: Supplementary file 1 [file Table_1.pdf]

Supplementary Table 1. Description of Each Beverage Group for Participants in the Diet, CKD, and APOL1 (DCA) Study (2021-2023)

Description	Beverage Group
Beer, European (>6% v/v alcohol)	Alcohol Beverage
Beer, European (4.6% v/v alcohol)	Alcohol Beverage
Beer, maize (ca. 3% v/v alcohol)	Alcohol Beverage
Beer, millet (ca. 3% v/v alcohol)	Alcohol Beverage
Beer, sorghum (ca. 3% v/v alcohol)	Alcohol Beverage
Palm wine (ca. 3.8% v/v alcohol)	Alcohol Beverage
Sap, palm, fresh (0.3% v/v alcohol)	Alcohol Beverage
Beer, European (>6% v/v alcohol)	Alcohol Beverage
Whiskey	Alcohol Beverage
Irish Cream	Alcohol Beverage
Coffee, instant, powder	Coffee
Coffee, liquid	Coffee
Milk, cow, whole, pasteurized or UHT, 3.5% fat	Milk and Milk products
Milk, cow, powder, whole, unfortified	Milk and Milk products
Breastmilk, mature	Milk and Milk products
Yoghurt, plain, from whole cow’s milk	Milk and Milk products
Milk, cow, reduced fat, 1.5–2% fat	Milk and Milk products
Milk, cow, skimmed, 0.5% fat	Milk and Milk products
Milk, cow, canned, condensed, sweetened	Milk and Milk products
Milk, cow, canned, evaporated	Milk and Milk products
Milk, cow, powder, skimmed, unfortified	Milk and Milk products
Breastmilk, colostrum	Milk and Milk products
Milk, cow, whole, fermented	Milk and Milk products
Yoghurt, plain, from reduced fat/skimmed cow’s milk	Milk and Milk products
Yoghurt, from reduced fat/skimmed cow’s milk, sweetened/with fruit	Milk and Milk products
Milk, cow, whole, raw, 4.5% fat	Milk and Milk products

Wheat drink	Milk and Milk products
Juice, lemon, fresh, unsweetened	Juice
Juice, orange, fresh, unsweetened	Juice
Juice, canned or bottled, sweetened (e.g. apple)	Juice
Juice, canned or bottled, unsweetened (e.g. grapefruit)	Juice
Water, tap	Other Beverages
Coconut water	Other Beverages
Soybean milk, fluid, from soybeans only, unfortified	Plant Milk/Drink
Kunu, Brukina, Tigernut drink	Plant Milk/Drink
Non-dairy milk	Plant Milk/Drink
Carbonated drink	Soda
Energy Drink	Soda
Carbonated drink	Soda
Energy Drink	Soda
Tea, infusion	Tea
Ovaltine beverage with whole milk, without sugar, fortified*	Tea
Ovaltine beverage with reduced fat milk, without sugar, fortified*	Tea
Ovaltine beverage with skimmed milk, without sugar, fortified*	Tea
Ovaltine powder, fortified	Tea
