# Supplementary material for: Beverage patterns, blood pressure, and proteinuria among West Africans with chronic kidney disease: a cross-sectional analysis of the diet, CKD, and apolipoprotein L1 study
Source: Front Nutr. 2026 Feb 6;13:1724375. doi: 10.3389/fnut.2026.1724375 (PMC12920209; doi:10.3389/fnut.2026.1724375)
Supplement: Supplementary file 2 [file Table_2.pdf]

Supplementary Table 2. Sensitivity Analysis: Association of Beverage Patterns with Systolic and Diastolic Blood Pressure and Proteinuria, Adjusted for Antihypertensive Medication Use, in Participants of the Diet, CKD, and APOL1 (DCA) Study (2021–2023)

	SBP*		DBP*		Proteinuria+	
	Model 1 [Estimate (95% CI)]	Model 2 [Estimate (95% CI)]	Model 1 [Estimate (95% CI)]	Model 2 [Estimate (95% CI)]	Model 1 [Estimate (95% CI)]	Model 2 [Estimate (95% CI)]
**Sugar-Sweetened Beverage & Alcohol Beverage Pattern**						
**Tertile 1**	Ref	Ref	Ref	Ref	Ref	Ref
**Tertile 2**	-0.55 (-5.06, 3.92)	1.33 (-2.76, 5.35)	0.31 (-2.68, 3.22)	0.15 (-2.80, 3.05)	0.12 (-0.16, 0.42)	0.05 (-0.23, 0.34)
**Tertile 3**	**-4.49 (-9.03, -0.04)**	-2.05 (-6.24, 2.03)	1.84 (-1.26, 4.74)	0.13 (-2.90, 3.07)	0.24 (-0.04, 0.54)	-0.02 (-0.30, 0.27)
**P for trend**	0.051	0.337	0.221	0.933	0.099	0.877
**Continuous**	-0.74 (-2.40, 0.90)	0.16 (-1.35, 1.64)	0.93 (-0.19, 1.99)	0.50 (-0.59, 1.56)	0.08 (-0.03, 0.19)	-0.01 (-0.11, 0.10)
**Milk and Alcohol Beverage Pattern**						
**Tertile 1**	Ref	Ref	Ref	Ref	Ref	Ref
**Tertile 2**	**6.55 (2.15, 10.95)**	3.59 (-0.47, 7.71)	1.57 (-1.31, 4.53)	1.34 (-1.57, 4.34)	0.12 (-0.17, 0.40)	0.14 (-0.15, 0.42)
**Tertile 3**	3.11 (-1.38, 7.55)	2.72 (-1.14, 6.56)	2.15 (-0.75, 5.12)	2.62 (-0.13, 5.45)	0.03 (-0.26, 0.32)	0.13 (-0.14, 0.40)
**P for trend**	0.173	0.174	0.151	0.069	0.823	0.339
**Continuous**	1.24 (-0.45, 2.91)	0.77 (-0.74, 2.26)	0.58 (-0.52, 1.68)	0.74 (-0.33, 1.83)	-0.02 (-0.13, 0.09)	0.02 (-0.09, 0.12)
**Sugar-Sweetened & Water Without Alcohol Beverage Pattern**						
**Tertile 1**	Ref	Ref	Ref	Ref	Ref	Ref
**Tertile 2**	2.89 (-1.54, 7.29)	1.69 (-2.21, 5.60)	-1.02 (-3.91, 1.90)	-0.37 (-3.16, 2.47)	-0.02 (-0.31, 0.26)	0.03 (-0.24, 0.30)
**Tertile 3**	-1.02 (-5.45, 3.36)	-1.54 (-5.42, 2.28)	-2.02 (-4.94, 0.86)	-1.89 (-4.67, 0.87)	-0.01 (-0.30, 0.27)	0.01 (-0.26, 0.28)
**P for trend**	0.649	0.438	0.172	0.189	0.953	0.934
**Continuous**	-0.09 (-1.80, 1.61)	0.51 (-0.99, 2.00)	-0.11 (-1.23, 1.01)	0.00 (-1.08, 1.07)	-0.02 (-0.13, 0.09)	-0.05 (-0.15, 0.06)
**Milk & Milk Products Beverage Pattern**						
**Tertile 1**	Ref	Ref	Ref	Ref	Ref	Ref
**Tertile 2**	2.91 (-1.71, 7.37)	**5.62 (1.57, 9.57)**	1.39 (-1.59, 4.31)	1.97 (-0.91, 4.87)	0.05 (-0.25, 0.34)	0.05 (-0.23, 0.33)

**Tertile 3**	-0.78 (-5.47, 3.74)	2.93 (-1.17, 6.89)	0.52 (-2.47, 3.49)	0.83 (-2.04, 3.75)	0.11 (-0.19, 0.41)	0.01 (-0.26, 0.29)
**P for trend**	0.737	0.159	0.733	0.58	0.456	0.925
**Continuous**	-0.54 (-2.42, 1.30)	0.31 (-1.30, 1.90)	0.43 (-0.76, 1.63)	0.22 (-0.92, 1.42)	0.05 (-0.08, 0.17)	-0.01 (-0.12, 0.10)

Model 1: included beverage pattern and the random effect (clinical site). Model 2: Adjusted for age, sex, education, income, antihypertensive medication *proteinuria, +hypertension, estimated Glomerular Filtration Rate (eGFR), Smoking, Diabetes, BMI, and Total Energy (per 1,000 KCAL). The participants received a score for each pattern based on their consumption and the PC scores were divided into 3 equal groups to facilitate comparisons. Tertile 3 represents the highest adherence to a specific beverage pattern, tertile 2 moderate, and tertile 1 the lowest.
