# Supplementary material for: Beverage patterns, blood pressure, and proteinuria among West Africans with chronic kidney disease: a cross-sectional analysis of the diet, CKD, and apolipoprotein L1 study
Source: Front Nutr. 2026 Feb 6;13:1724375. doi: 10.3389/fnut.2026.1724375 (PMC12920209; doi:10.3389/fnut.2026.1724375)
Supplement: Supplementary file 3 [file Table_3.pdf]

Supplementary Table 3. Association Between Beverage Groups and Systolic Blood Pressure for Participants in the Diet, CKD, and APOL1 (DCA) Study (2021-2023)

**Beverage Group**	**Model 1 [Estimate (95% CI)]**	**Model 2 [Estimate (95% CI)]**
Alcohol Beverage	-0.09 [-1.91, 1.70]	-0.50 [-2.32, 1.29]
Dairy milk & its products	-0.45 [-2.33, 1.40]	0.32 [-1.37, 2.00]
Soda	-1.91 [-3.72, -0.12]	-0.74 [-2.3, 0.83]
Tea	0.16 [-1.66, 2.02]	-1.17 [-2.84, 0.52]
Coffee	-1.41 [-3.19, 0.41]	-1.43 [-2.95, 0.12]
Juice	-0.70 [-2.49, 1.11]	-0.34 [-1.90, 1.22]
Other Beverages	-0.59 [-2.43, 1.30]	0.23 [-1.36, 1.83]
Plant Milk/Drink	0.19 [-1.62, 2.01]	-0.23 [-1.83, 1.37]

Model 1: included only the beverage group of interest and the random effect (clinical site). Model 2: fully adjusted model including age, sex, education, income, proteinuria, baseline eGFR, smoking, diabetes, BMI, and total energy intake.
