# Supplementary material for: Beverage patterns, blood pressure, and proteinuria among West Africans with chronic kidney disease: a cross-sectional analysis of the diet, CKD, and apolipoprotein L1 study
Source: Front Nutr. 2026 Feb 6;13:1724375. doi: 10.3389/fnut.2026.1724375 (PMC12920209; doi:10.3389/fnut.2026.1724375)
Supplement: Supplementary file 4 [file Table_4.pdf]

Supplementary Table 4. Baseline Characteristics of Participants Across Tertiles of Beverage Patterns in the Diet, CKD, and APOL1 (DCA) Study

Characteristic	N = 494*
**Age (years)**	49 (18)
**BMI (Kg/m^2)**	26.3 (5.7)
**Female**	237 (48%)
**Education[2]**	
Secondary/less	345 (70%)
Higher	148 (30%)
**Income**	
Low/Medium	268 (54%)
High	24 (4.9%)
**Smoking+**	28 (5.8%)
**Drinking+**	207 (43%)
**Antihypertensive Medication**	351 (71%)
**SBP mm Hg [Mean (SD)]**	130 (21)
**Diabetes**	102 (21%)
**eGFR, CKD-EPI 2009, mL/min/$1.73m^2$**	67 (38.78, 98)
**Proteinuria, mg**	0.34 (0.16, 1.12)

eGFR, CKD-EPI 2009- estimated Glomerular Filtration Rate, Chronic Kidney Disease Epidemiology Collaboration 2009 not corrected for race. SBP- systolic blood pressure.

* Mean (SD); n (%); Median (Q1, Q3).

+ Missing observations: 1 for Education, 10 for Smoking, and 11 for Drinking; 202 Don’t wish to answer for Income.
